# Supplementary material for: Analysis of PPARγ Signaling Activity in Psoriasis
Source: Int J Mol Sci. 2021 Aug 10;22(16):8603. doi: 10.3390/ijms22168603 (PMC8395241; doi:10.3390/ijms22168603)
Supplement: Supplementary file 1 [file ijms-22-08603-s001.zip › Supplemental materials_Analysis of PPARg signaling activity in psoriasis/Pathway models/Models images and html files/Interleukin-17 and interleukin-22 signaling in psoriasis/142318.html]

keratinocyte adhesion


# Cell Process keratinocyte adhesion

|  |  |
| --- | --- |
| URN | urn:agi-cellproc:keratinocyte%20adhesion |
| Total Entities | 0 |
| Connectivity | 138 |
| Name | keratinocyte adhesion |

---

|  |  |
| --- | --- |
| Pathway | 2\_Interleukin-17 and interleukin-22 signaling in psoriasis |
|  | Interleukin-17 and interleukin-22 signaling in psoriasis |

---

|  |  |
| --- | --- |
| MedScan ID | 4000192 |

---

|  |  |
| --- | --- |
| Alias | keratocyte adherence |
|  | keratinocytic adherence |
|  | keratinocyte adhesion |
|  | keratocytic attachment |
|  | keratinocyte adherence |
|  | keratocytic adherence |

---
